# Heart Failure Progression in the Setting of Persistent Atrial Fibrillation, Frequent Premature Ventricular Complexes, and Malignancy‐Related Limitations to Guideline‐Directed Medical Therapy: A Case Report

**DOI:** 10.1002/ccr3.73771

**Published:** 2026-10-21

**Authors:** Douni Roger, Waleed Shaker, Majid Yavari, Rajan Thakur, Mark Castellani, Zan Siddiqi, Maurice Tiotsop

**Affiliations:** ^1^ Michigan State University East Lansing Michigan USA; ^2^ University of Louisville School of Medicine Louisville Kentucky USA

**Keywords:** arrhythmia‐induced cardiomyopathy, atrioventricular nodal ablation, cardiac resynchronization therapy, heart failure with reduced ejection fraction, persistent atrial fibrillation, premature ventricular complexes

## Abstract

Persistent atrial fibrillation and frequent premature ventricular complexes may contribute to worsening heart failure, particularly when malignancy limits guideline‐directed medical therapy. In patients with progressive ventricular dysfunction and limited pharmacologic options, atrioventricular nodal ablation with cardiac resynchronization therapy‐defibrillator implantation may provide durable rate control and support subsequent ventricular functional recovery.

## Graphical abstract

Persistent atrial fibrillation, frequent premature ventricular complexes, and limited tolerance of guideline‐directed medical therapy contributed to progressive ventricular dysfunction, with LVEF declining from approximately 50% to 20%–25%. AV nodal ablation with CRT‐D implantation provided durable ventricular rate control and biventricular pacing. At 3‐month follow‐up, LVEF improved to 35%–40%, symptoms stabilized, and the LifeVest was discontinued.

Key takeaway: When pharmacologic rate control and GDMT are limited, AV nodal ablation with CRT‐D may provide durable rate control and support ventricular recovery.

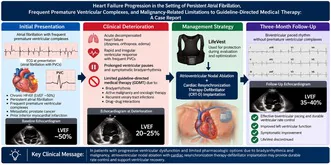

## 1 Introduction

Arrhythmia‐induced cardiomyopathy (AiCM) has emerged as an important and potentially reversible contributor to left ventricular (LV) dysfunction, particularly when arrhythmia burden is significant [1]. Recent consensus emphasizes that AiCM should be suspected in patients with persistent atrial fibrillation (AF), mean ventricular rates exceeding 100 bpm, or high premature ventricular contraction (PVC) burdens (generally > 10%), as these factors may independently and synergistically impair ventricular function through tachycardia, irregularity, and mechanical dyssynchrony [2, 3]. While timely arrhythmia control often leads to functional recovery, real‐world management is frequently complicated—up to half of patients may be unable to tolerate full guideline‐directed medical therapy (GDMT) even in standard heart failure settings [1, 4, 5]. These challenges are further compounded in patients with malignancy, where cardiotoxic cancer treatments, drug–drug interactions, and heightened infection risk may necessitate down‐titration or discontinuation of key heart failure (HF) therapies [2]. In such complex clinical scenarios, incorporating individualized, device‐based strategies may be vital to stabilizing patient status and preserving myocardial function.

We report a case of persistent AF with frequent PVCs and progressive heart failure with reduced ejection fraction (HFrEF) in the setting of active prostate cancer, illustrating the therapeutic limitations of pharmacologic therapy and the successful use of atrioventricular (AV) nodal ablation with cardiac resynchronization therapy‐defibrillation (CRT‐D) implantation.

## 2 Case Presentation/Examination

A 76‐year‐old man with chronic heart failure with reduced ejection fraction (HFrEF), persistent atrial fibrillation (AF), metastatic prostate cancer, hypertension, hyperlipidemia, and a history of inferior myocardial infarction presented with progressive dyspnea, orthopnea, and bilateral lower‐extremity edema. On admission, vital signs revealed a blood pressure of 138/76 mmHg, an irregular heart rate of 96 beats/min, respiratory rate of 20 breaths/min, oxygen saturation of 94% on room air, and temperature of 36.8°C. Physical examination demonstrated elevated jugular venous pressure to the angle of the mandible at 45°, bibasilar crackles, an irregularly irregular rhythm with intermittent tachycardia, and a Grade II/VI systolic murmur at the apex. Bilateral 1+ pitting edema was present to the mid‐shin. The initial electrocardiogram demonstrated persistent atrial fibrillation with frequent premature ventricular complexes (Figure 1). Findings were consistent with acute decompensated heart failure.

**FIGURE 1:**
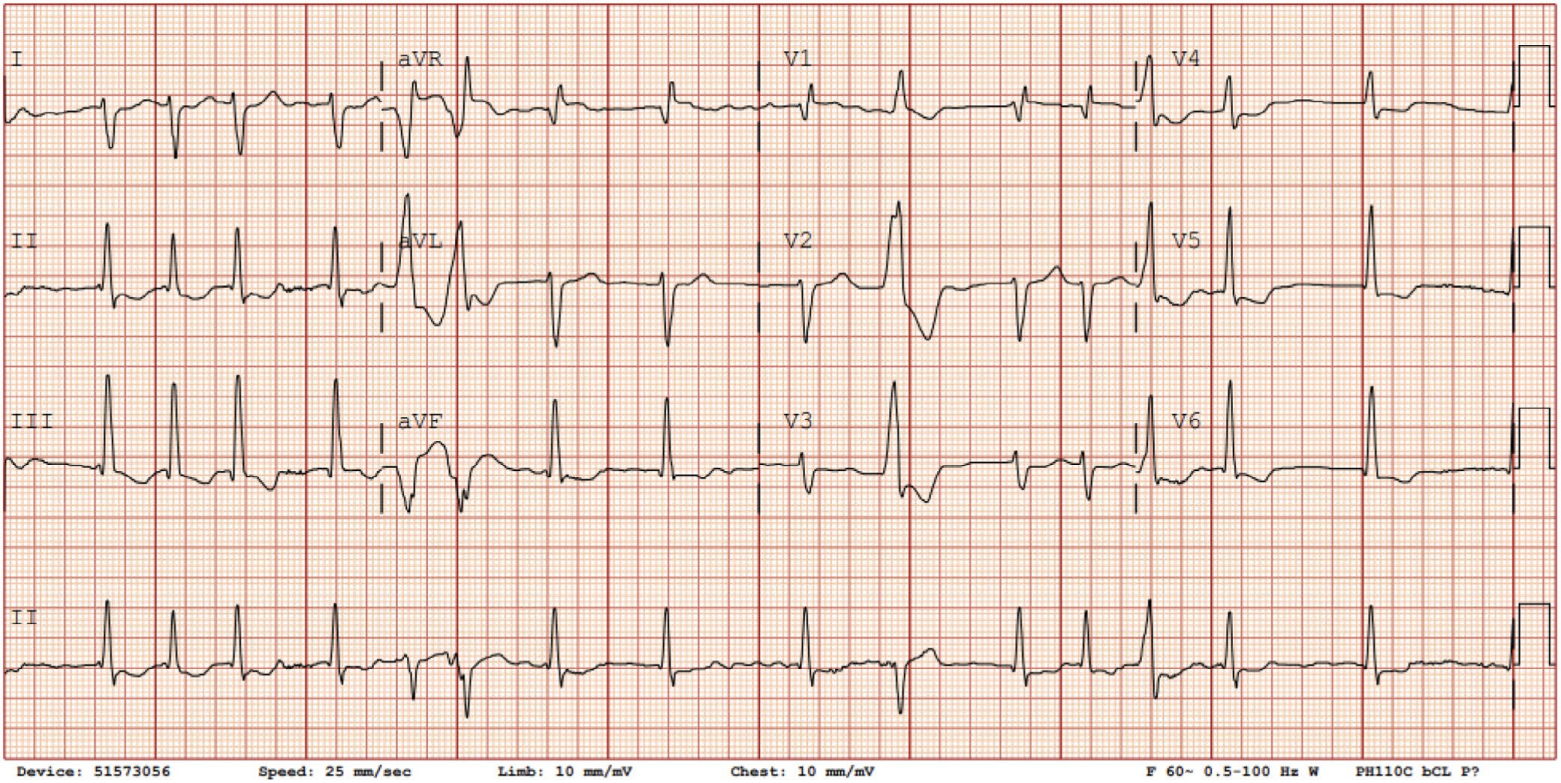
Electrocardiogram demonstrating atrial fibrillation with frequent premature ventricular complexes and ventricular pauses.

## 3 Methods

Initial laboratory evaluation (Table 1) demonstrated elevated brain natriuretic peptide, mild troponin elevation consistent with demand ischemia, and a subtherapeutic international normalized ratio. Urinalysis revealed pyuria, and urine culture grew *Proteus mirabilis* sensitive to ceftriaxone.

**TABLE 1:** Initial laboratory findings.

Test	Patient value	Normal range	Interpretation
Troponin	167 ng/L	< 0.04 ng/L	Elevated
INR	1.6	2.0–3.0 (on warfarin)	Subtherapeutic
Creatinine (Cr)	1.2 mg/dL	~0.6–1.3 mg/dL	At baseline; stable renal function
BUN	28 mg/dL	7–20 mg/dL	Mildly elevated; reflect volume status
BNP	2866 pg/mL (prior)	< 100 pg/mL	Severely elevated—consistent with acute decompensated heart failure (previous 198 pg/mL)
Sodium	149 mmol/L	135–145 mmol/L	Hypernatremia – dehydration
White blood count (WBC)	12.5 × 10^9^/L	4.0–11.0 × 10^9^/L	Leukocytosis – suspected infection

*Note:* Reference ranges may vary slightly between laboratories.

The differential diagnosis for the worsening left ventricular systolic dysfunction included arrhythmia‐mediated cardiomyopathy related to persistent atrial fibrillation and ventricular ectopy, ischemic cardiomyopathy related to the patient's prior myocardial infarction, and other potentially reversible contributors such as obstructive sleep apnea. Pharmacologic stress imaging demonstrated a large fixed inferior and inferolateral perfusion defect without inducible ischemia, supporting a prior ischemic substrate without evidence of active inducible ischemia.

Continuous inpatient telemetry demonstrated persistent atrial fibrillation with fluctuating ventricular rates, frequent premature ventricular complexes, and prolonged ventricular pauses lasting up to 10 s. The pauses were interpreted as intermittent failure of atrioventricular conduction of the rapid, irregular atrial impulses during persistent atrial fibrillation rather than sinus pauses. The available telemetry documentation did not provide a quantitative PVC burden or PVC frequency per unit time. Transthoracic echocardiography revealed severe left ventricular (LV) systolic dysfunction with an EF of 20%–25%, global hypokinesis, and marked left atrial dilation (6.1 cm). The right ventricle was mildly dilated with preserved systolic function. A prior echocardiogram obtained from an outside hospital approximately 1 year earlier had demonstrated moderately reduced LV function (ejection fraction 50%). Pharmacologic nuclear stress testing demonstrated a large, fixed inferior and inferolateral perfusion defect consistent with prior infarction, unchanged from a 2022 study, without evidence of inducible ischemia (Figure 2). Guideline‐directed medical therapy was substantially influenced by several competing clinical factors. Carvedilol was withheld because of prolonged ventricular pauses and symptomatic bradyarrhythmia. Spironolactone was avoided because of potential interactions with ongoing oncologic therapy, while the sodium‐glucose cotransporter‐2 inhibitor was discontinued because of recurrent urinary tract infections. Following clinical stabilization, sacubitril/valsartan (Entresto), hydralazine‐isosorbide dinitrate, and warfarin were resumed prior to discharge. Additional medications with potential interactions with chemotherapy were withheld in close coordination with the oncology team, limiting full optimization of guideline‐directed medical therapy during hospitalization.

**FIGURE 2:**
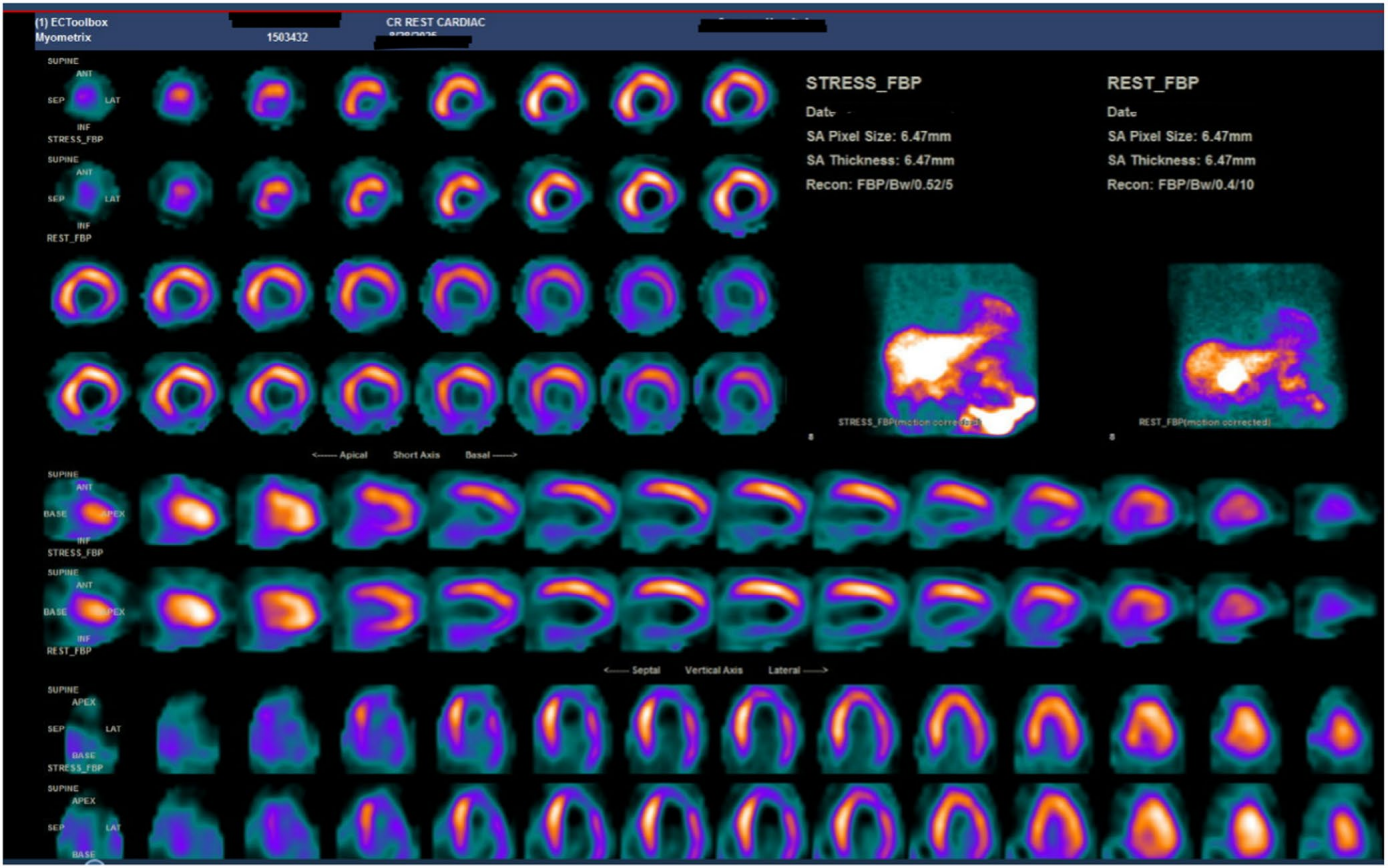
Stress test images with perfusion show inferior and inferolateral defect.

Given the newly documented severe reduction in LVEF from approximately 50% on echocardiography 1 year earlier to 20%–25% during the current hospitalization, a wearable cardioverter‐defibrillator (LifeVest) was prescribed as temporary protection while definitive device‐based therapy was being planned. After persistent atrial fibrillation with rapid ventricular response, prolonged ventricular pauses, and limited tolerance of pharmacologic rate‐control therapy, the patient underwent atrioventricular nodal ablation with CRT‐D implantation during the same hospitalization. After successful atrioventricular nodal ablation and biventricular CRT‐D implantation (Figure 3), the patient was discharged with plans for close outpatient cardiology follow‐up and further optimization of heart failure therapy as tolerated.

**FIGURE 3:**
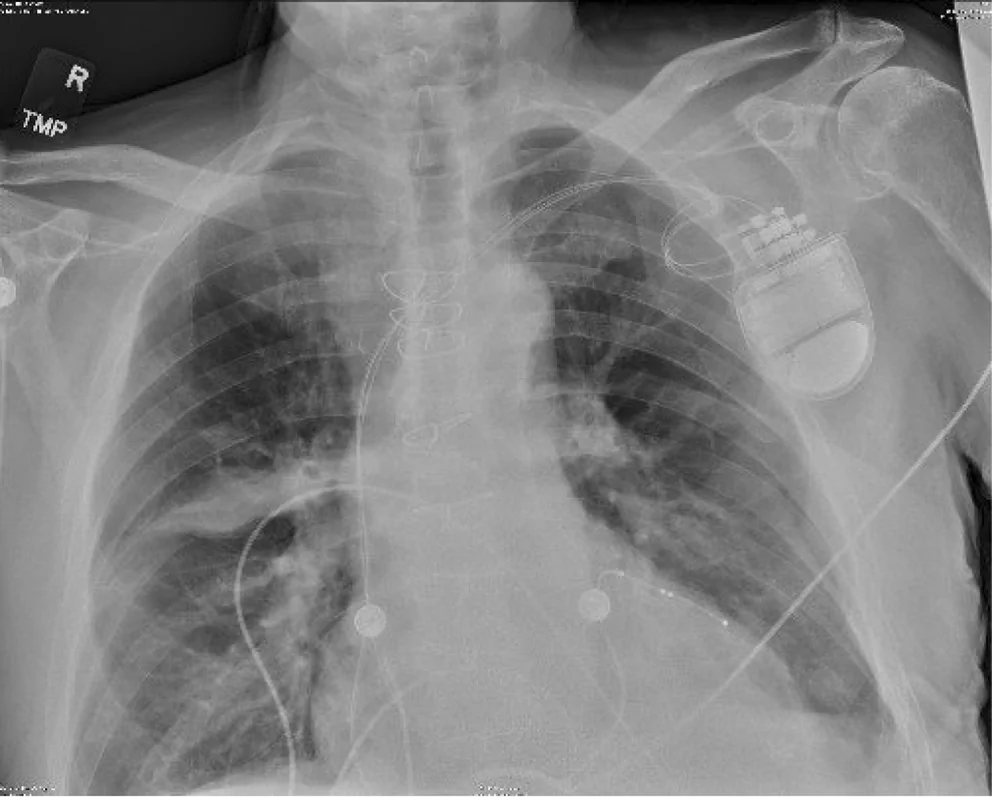
Postoperative chest radiograph demonstrating appropriate positioning of the CRT‐D leads following successful implantation.

## 4 Conclusion and Results

Following AV nodal ablation, biventricular pacing achieved effective and durable ventricular rate control. The patient experienced stabilization of heart failure symptoms and reduction in arrhythmia burden prior to discharge. He was discharged in stable condition after a 2‐week hospitalization. At discharge, the patient remained clinically stable on a tailored heart failure regimen (Table 2) that included resumed sacubitril/valsartan, hydralazine‐isosorbide dinitrate, and warfarin, with carvedilol and mineralocorticoid receptor antagonist therapy deferred because of persistent bradyarrhythmia and oncologic treatment considerations. Ongoing optimization of guideline‐directed medical therapy was planned during outpatient follow‐up as tolerated.

**TABLE 2:** Medication management during hospitalization and rationale for treatment decisions.

Medication	Status	Reason
Carvedilol	Held	Prolonged ventricular pauses and symptomatic bradyarrhythmia
Sacubitril/Valsartan	Resumed before discharge	Clinical stabilization permitted re‐initiation
Hydralazine/Isosorbide dinitrate	Resumed before discharge	Continued after stabilization
Spironolactone	Not initiated	Potential interaction with ongoing chemotherapy
SGLT2 inhibitor	Held	Recurrent urinary tract infections
Warfarin	Continued/Resumed	Stroke prevention in persistent atrial fibrillation
Loop diuretic	Continued	Volume management

At the 2‐week and 3‐month follow‐up visits, the patient demonstrated sustained ventricular rate control, stabilization of heart failure symptoms, and overall clinical improvement. Repeat transthoracic echocardiography at 3 months showed improvement in left ventricular ejection fraction from 20%–25% to 35%–40%. Given the improvement in ventricular function, the wearable cardioverter‐defibrillator was discontinued after the 3‐month follow‐up. A standard 12‐lead electrocardiogram obtained at the 3‐month follow‐up demonstrated a biventricular paced rhythm without premature ventricular complexes during the recording (Figure 4). Because this was a single brief recording, the absence of PVCs on this ECG does not establish resolution or quantify a reduction in ventricular ectopy.

**FIGURE 4:**
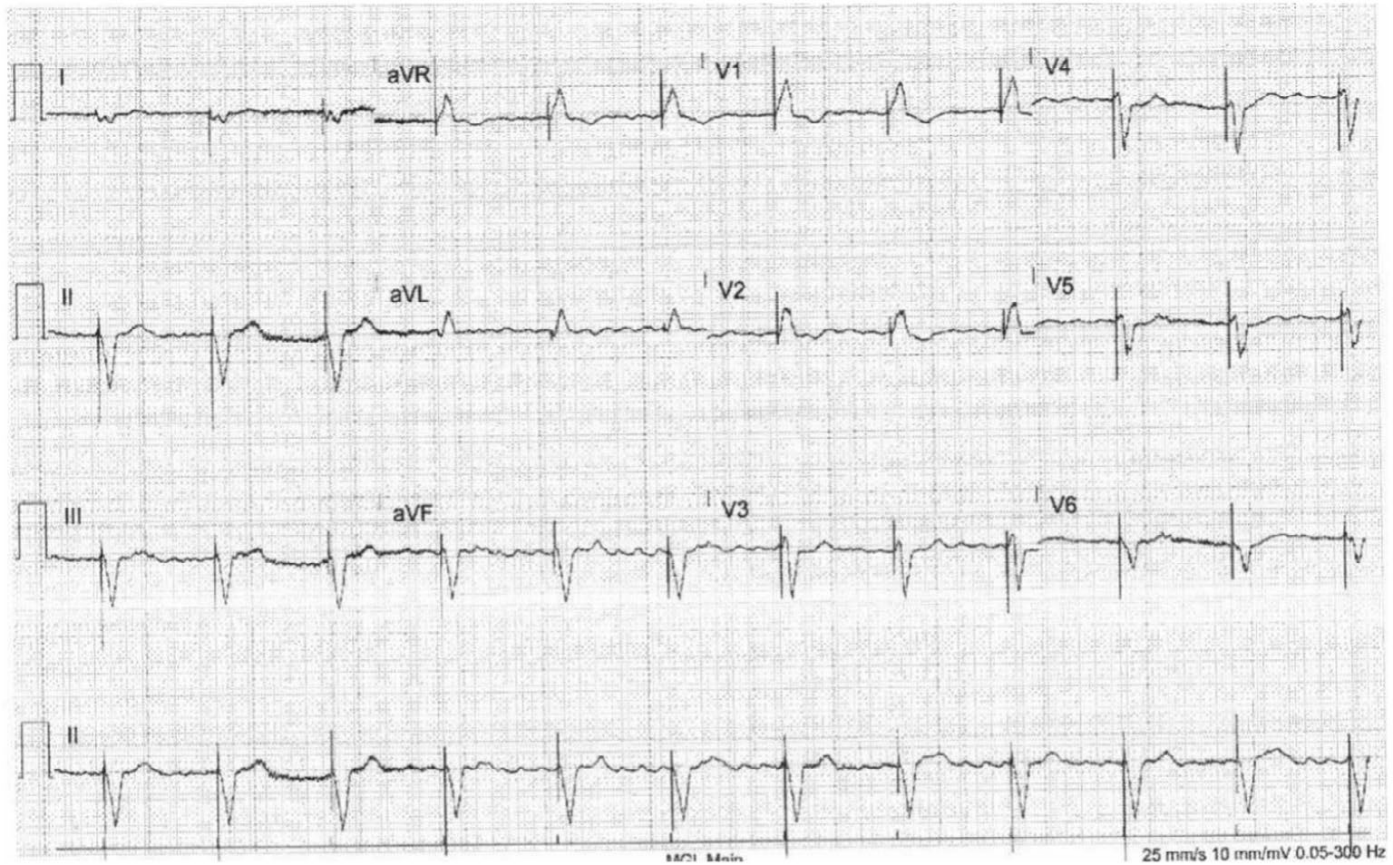
Twelve‐lead electrocardiogram obtained at the 3‐month follow‐up demonstrating biventricular paced rhythm without premature ventricular complexes during the recording.

## 5 Discussion

This case illustrates the complex interaction between persistent atrial fibrillation, frequent PVCs, and cardiomyopathy in the progression of HFrEF. Persistent AF is common in patients with reduced LV function and is associated with increased hospitalization and mortality [6]. Uncontrolled ventricular rates may accelerate tachycardia‐mediated cardiomyopathy and exacerbate LV dysfunction [7].

PVC‐induced cardiomyopathy is well recognized, particularly when PVC burden is sufficiently high. In patients with pre‐existing ischemic cardiomyopathy, ventricular ectopy may also contribute to functional decline [8]. In this case, frequent PVCs were observed on continuous inpatient telemetry; however, no quantitative PVC burden or frequency per unit time was documented, and formal ambulatory monitoring was not performed. Therefore, PVC‐induced cardiomyopathy cannot be established or reasonably inferred as the primary cause of the decline in LV function. The ventricular ectopy is more appropriately considered a potential contributor to arrhythmia‐mediated worsening of established cardiomyopathy. Pharmacologic management remains first‐line therapy; however, intolerance to GDMT frequently limits optimal treatment. In this patient, malignancy‐related considerations precluded use of several cornerstone therapies, creating a therapeutic gap that necessitated a device‐based strategy.

Atrioventricular (AV) nodal ablation with biventricular pacing has emerged as an effective option for patients with symptomatic AF and HFrEF who are unable to tolerate or fail conventional therapies. The APAF‐CRT trial demonstrated that AV nodal ablation combined with cardiac resynchronization therapy (CRT) significantly reduced mortality compared to pharmacologic rate control in patients with permanent AF and narrow QRS HFrEF [9]. This approach provides durable rate control, prevents tachycardia‐induced worsening of LV function, and optimizes resynchronization in patients with dilated atria and refractory arrhythmia.

Obstructive sleep apnea represents another potentially reversible contributor to HFrEF. Although formal sleep evaluation was not performed during hospitalization because acute management of decompensated heart failure and refractory arrhythmia was prioritized, outpatient assessment was recommended when clinically appropriate.

Coronary angiography was considered during hospitalization given the patient's severe left ventricular systolic dysfunction. However, pharmacologic stress imaging demonstrated a large, fixed inferior and inferolateral perfusion defect without evidence of inducible ischemia, consistent with prior infarction. Given his advanced age, metastatic malignancy, multiple comorbidities, elevated procedural risk, and the family's preference to avoid invasive testing, coronary angiography was deferred after multidisciplinary discussion. This decision was not expected to alter the immediate management strategy.

A prolonged external loop recorder was not pursued because continuous inpatient telemetry had already documented the clinically relevant arrhythmias, and additional ambulatory monitoring was not expected to alter therapeutic decision‐making. Cardiac magnetic resonance imaging was not obtained because the patient had a well‐established ischemic substrate, persistent atrial fibrillation, and a clear clinical indication for cardiac resynchronization therapy‐defibrillator implantation. Additional tissue characterization was therefore unlikely to alter management.

A diagnostic electrophysiology study was not performed because continuous inpatient telemetry had already demonstrated persistent atrial fibrillation with rapid ventricular response, frequent premature ventricular complexes, and prolonged ventricular pauses. The electrophysiology team concluded that additional invasive diagnostic testing would not alter management, as the patient already met clinical indications for atrioventricular nodal ablation with cardiac resynchronization therapy‐defibrillator implantation. No procedural image from a diagnostic electrophysiology study is available because a formal diagnostic EP study was not performed. Left bundle branch area pacing (LBBAP) was considered during procedural planning. However, biventricular cardiac resynchronization therapy (CRT) remains the established and guideline‐supported approach for patients with heart failure with reduced ejection fraction undergoing atrioventricular nodal ablation at our institution [10]. Given the extensive clinical experience with CRT, the patient's underlying ischemic cardiomyopathy, and institutional practice patterns, CRT‐D implantation was selected. Recent physiologic pacing guidelines recognize LBBAP as an emerging alternative in appropriately selected patients.

The patient's markedly dilated left atrium, persistent AF with rapid ventricular response, and limited tolerance of GDMT supported the decision to proceed with AV nodal ablation and CRT‐D implantation. Frequent ventricular ectopy was observed on continuous telemetry and may have contributed to progressive LV dysfunction; however, its quantitative burden could not be determined. A wearable cardioverter‐defibrillator (LifeVest) was prescribed during the hospitalization as temporary protection while definitive device therapy was being planned. The LifeVest was continued after discharge and discontinued after 3‐month follow‐up when echocardiography demonstrated improvement in LVEF to 35%–40%.

## 6 Limitations

Several limitations should be acknowledged. First, the patient had pre‐existing ischemic and valvular heart disease, making it difficult to attribute the decline in left ventricular systolic function solely to arrhythmia‐induced cardiomyopathy. Accordingly, this case is more appropriately interpreted as arrhythmia‐mediated worsening of established cardiomyopathy. Second, frequent premature ventricular complexes were identified qualitatively on inpatient telemetry, but no quantitative PVC burden or frequency per unit time was documented. Formal ambulatory Holter monitoring and pre‐intervention device interrogation were not performed, limiting assessment of the magnitude of ventricular ectopy before and after intervention. Third, the coexistence of acute infection, metastatic prostate cancer, and oncology‐related restrictions to guideline‐directed medical therapy introduced important confounding factors that likely contributed to heart failure progression. Fourth, advanced imaging with cardiac magnetic resonance imaging was not performed because it was not expected to alter management in the setting of known ischemic cardiomyopathy, persistent atrial fibrillation, and an established indication for cardiac resynchronization therapy‐defibrillator implantation. Quantitative device interrogation data, including the percentage of biventricular pacing, were not available for inclusion. Additionally, formal ambulatory Holter or external loop recorder monitoring, quantitative device interrogation data, and intraprocedural images from the electrophysiology procedure and AV nodal radiofrequency ablation were not available. Therefore, the burden of ventricular ectopy and the percentage of biventricular pacing could not be quantified beyond the available inpatient telemetry and follow‐up electrocardiographic findings.

Despite these limitations, this case illustrates how multiple converging clinical factors—including persistent atrial fibrillation, ventricular ectopy, cardiomyopathy, active malignancy, and restricted guideline‐directed medical therapy—may accelerate heart failure progression. It also highlights the important role of atrioventricular nodal ablation with cardiac resynchronization therapy in achieving durable rate control and improving ventricular function when conventional medical therapy is limited.

## Author Contributions

**Douni Roger:** conceptualization, data curation, investigation, resources. **Waleed Shaker:** formal analysis, investigation, resources. **Majid Yavari:** supervision, validation. **Rajan Thakur:** resources, supervision, validation. **Mark Castellani:** resources, supervision. **Zan Siddiqi:** investigation, resources, supervision, writing – review and editing. **Maurice Tiotsop:** project administration, writing – review and editing.

## Funding

The authors have nothing to report.

## Consent

Written informed consent was obtained from the patient for the publication of this case report and any accompanying images or clinical information. The patient was informed that all reasonable efforts would be made to ensure anonymity and that no identifying information would be disclosed.

## Conflicts of Interest

The authors declare no conflicts of interest.

## Data Availability

All data relevant to this case report are included within the article. No additional datasets were generated or analyzed during the current study.
